# Maintenance of Zebrafish Lines at the European Zebrafish Resource Center

**DOI:** 10.1089/zeb.2015.1205

**Published:** 2016-07-01

**Authors:** Robert Geisler, Nadine Borel, Marco Ferg, Jana Viktoria Maier, Uwe Strähle

**Affiliations:** Institute of Toxicology and Genetics, Karlsruhe Institute of Technology (KIT), Eggenstein-Leopoldshafen, Germany.

## Abstract

We have established a European Zebrafish Resource Center (EZRC) at the KIT. This center not only maintains and distributes a large number of existing mutant and transgenic zebrafish lines but also gives zebrafish researchers access to screening services and technologies such as imaging and high-throughput sequencing, provided by the Institute of Toxicology and Genetics (ITG). The EZRC maintains and distributes the stock collection of the Nüsslein-Volhard laboratory, comprising over 2000 publicly released mutations, as frozen sperm samples. Within the framework of the ZF-HEALTH EU project, the EZRC distributes over 10,000 knockout mutations from the Sanger Institute (United Kingdom), as well as over 100 mutant and transgenic lines from other sources. In this article, we detail the measures we have taken to ensure the health of our fish, including hygiene, quarantine, and veterinary inspections.

## Introduction

Due to its small size, easy breeding, and transparent embryos, the zebrafish are increasingly popular as a model organism for biomedical research, currently in use by over 360 European laboratories.^*^ The zebrafish are particularly useful for high-throughput approaches not feasible in rodents (chemical and genetic screening). These have resulted in the production of several thousand mutant and transgenic zebrafish lines in Europe alone.^1^ However, before the establishment of the EZRC, no permanent repository was available for these lines. Shipping to and from the only existing stock center (the Zebrafish International Resource Center, ZIRC, in the United States) is too expensive for many users.

With the EZRC, we fulfill this demand. We provide European zebrafish laboratories with easy and cost-effective access to the fish lines that they require for their research. We mirror popular lines of ZIRC and accept submissions from laboratories who want to make their own fish lines publicly available. Since already existing mutants do not have to be generated anew, this service also helps to achieve the goal of reducing animal experimentation. A current focus is on distributing mutant lines from an ongoing genome-wide knockout experiment at the Wellcome trust Sanger Institute.^2^

## Infrastructure

**FIG. 1.**
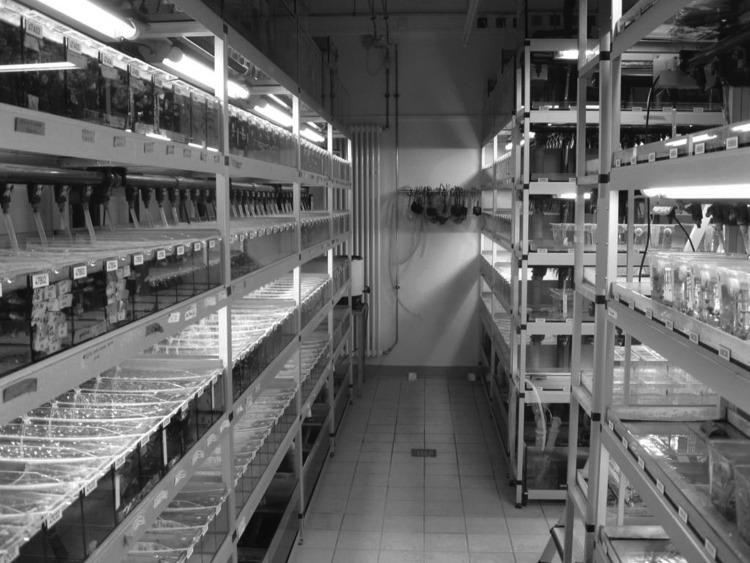
Quarantine room.

The European Zebrafish Resource Center uses space in two different buildings, several hundred meters apart, which helps to keep the core of the resource center free from contamination. All fish to be imported are kept in a Quarantine Room (shown in Fig. 1) and only their offspring are transferred as bleached eggs to the Core Fish Room in the main fish facility, which also houses a laboratory for genotyping, sperm freezing, and *in vitro* fertilization procedures, as well as shipment of stocks. Fish to be used for experimental purposes are transferred from the Core Fish Room to one of several experimental fish room, and are never returned after use.

Sperm freezing and *in vitro* fertilization (IVF) are essential for the EZRC since we store most of the lines as frozen sperm. Our methods are based on those used at the Sanger Institute.^3^ This protocol has the advantage of avoiding the use of milk powder, which is difficult to procure in consistent quality. Three cryogenic freezers are now in operation. The freezers are equipped with nitrogen tanks that are kept full for automatic emergency cooling and connected to the campus alarm center, which will alert EZRC personnel in case of an emergency. Two of these are located at the fish facility, a third one, for backup samples, is located in the building housing quarantine and screening facilities.

As a central element of our efforts, the Resource Center Database (RCDB) supports laboratory management of the resource center and provides biosafety records required by authorities. Commercial software for zebrafish laboratories was found to be lacking the special features required for the EZRC, and there was concern about the long-term safekeeping of EZRC data, if stored by a commercial provider. Therefore, an in-house software development was initiated using open-source tools (Linux, Apache, MySQL, and PHP). The database centers around the concept of stock records that describe the fish in one tank or the sperm samples in one CryoTube, and is never deleted. Based on this information, the software provides functionality for the graphical display of pedigrees. Each stock has events associated with it, which provide a log of its life history. Published alleles are automatically annotated with relevant information from the public ZFIN database. All fish tanks receive bar code labels printed from the database. We are currently looking to employ a new database developer who will be tasked with implementing additional features (e.g., genotyping and integration of mobile devices). Since our database software is tailored to our specific requirements as a stock center, it is not likely to be immediately useful for other fish facilities. However, as soon as our code is sufficiently stable and clean, we will be willing to make it available to other researchers as a basis for their own software development.

The public website of the EZRC is based on KIT's Content Management System, OpenText, and uses text files exported from the database (http://ezrc.kit.edu). This separation minimizes security risks and simplifies changes to both sites while development is ongoing. For each order, the site automatically generates a Purchase Order form, Material Transfer Agreement, and Biosafety Declaration form, which must be signed by the appropriate authorized officials at the customer's institution. The Biosafety Declaration is required for orders containing plasmids or transgenic zebrafish lines, to certify that the legal requirements for handling of genetically modified organisms at the recipient institute are met.

To assure consistent quality, standard operating procedures (SOPs) have been established for all major laboratory and husbandry tasks (Table 1). These operating procedures follow established standards as laid out in the Zebrafish Book^4^ and by the EuFishBioMed network (www.eufishbiomed.kit.edu/downloads/Guidelines_letter_English.pdf).

**Table 1.** List of SOPs in Force at the EZRC

*Title*	*Contents*
General	Good work practices, handling of mouse cages, artemia tanks, gloves, nets, tank covers and dishwashers, water changes, and health checks
Hygiene	Personal hygiene measures (see main text)
Routine operations	Daily and weekly schedule for fish room cleaning and maintenance
Artemia raising	Protocol for raising of artemia in 40 L barrels
Student employment	Rules for undergraduates employed in the fish facility
Bleaching of fish	Laboratory protocol (see main text)
Health monitoring	Schedule, scope, and number of fish to be examined
Fulfillment of orders	Weekly schedule for administrative tasks, IVF/crossing, and shipment in response to orders received
IVF and cryopreservation	Laboratory protocols (discussed in the main text)

EZRC, European Zebrafish Resource Center; IVF, *in vitro* fertilization; SOP, standard operating procedure.

## Stocks

The EZRC welcomes submission of fish lines and plasmids that laboratories want to make available for public distribution. Fish lines are accepted only as eggs disinfected with bleach, after completion of a submission form. Moreover, all submitted mutant, transgenic, and wild-type lines must have a ZFIN record and approved nomenclature. Submitted lines are grown up in quarantine for one generation and then their offspring are transferred to the core fish room.

The EZRC has received the entire collection of mutants from the three large-scale mutagenesis screens of Christiane Nüsslein-Volhard (Max Planck Institute for Developmental Biology, Tübingen). These lines were imported as entire containers with frozen sperm to minimize risk of transport. IVF of the Tübingen samples was tested with very good success. Shipments are made directly from IVF of these sperm samples. In total, the EZRC offers 1047 alleles from the Tübingen I screen, 55 alleles from the Tübingen 2000 screen, and 970 alleles from the Tübingen EU screen.

Within the framework of the ZF-HEALTH EU project, the EZRC and ZIRC agreed to take over distribution of the knockout lines generated by the TILLING method in the genome-wide Zebrafish Mutation Project of the Sanger Institute (Hinxton, United Kingdom).^2^ This agreement specifies that we receive two sperm samples of each sequenced F1 fish, while three go to ZIRC and three are retained by the Sanger Institute as a backup. The EZRC sends embryos either directly from an IVF with the F1 sperm samples (if the IVF yields a large number of embryos) or from crosses of the F2 generation (from a group mating or from identified carriers, if the number of F2 fish is too low for such a mating). Because only one sample and one backup are received from Sanger, an F2 and F3 generation is grown up for every line to generate additional sperm samples and then they are discarded again to save tank space. Ten thousand two hundred seventy-nine alleles from the Sanger Institute are now available from the EZRC, representing approximately one-third of all protein-coding zebrafish genes. Each F1 sperm sample contains 10–15 putative KO mutations.

Furthermore, the EZRC currently offers five wild-type stocks, 14 additional mutant lines, and 104 transgenic lines generated at the KIT or submitted by diverse laboratories, including 41 enhancer-trap lines from Vladimir Korzh (IMCB).^5^ The AB wild-type line of ZIRC is also used for all our IVFs and outcrosses and bred in large numbers for that purpose.

All providers of lines have licensed us to ship their lines under a standard Material Transfer Agreement (MTA) modeled on that of ZIRC. It requires the recipient to acknowledge both KIT and the lines’ provider, prohibits commercial use and redistribution, but contains no further restrictions. Commercial recipients must negotiate a license agreement directly with the provider. To recover our direct costs, we charge for shipment of fish and plasmids on a scale comparable to ZIRC (www.ezrc.kit.edu/downloads/EZRC_Pricing_Info.pdf).

We currently ship ∼30 zebrafish lines per month. The time from order to shipment is ∼12 months for alleles from Sanger F1 sperm samples. This is due to two facts: there is currently a backlog of orders due to high demand, and we need to grow up these alleles to generate additional sperm samples, making it impossible to ship them directly from the IVF. Shipping time is much less for other lines, which we can ship directly from a crossing or from an IVF (including Sanger lines for which we have already generated additional sperm samples).

## Aquaculture

The EZRC aquarium facilities use recirculating water systems (Aqua Schwarz, Göttingen and Müller + Pfleger, Rockenhausen) in every fish room. Water is pumped into storage tanks. These tanks are located above the aquaria. Through gravity, water is distributed into the aquarium tanks, which are equipped with an overflow system that directs water to the filter systems below the tanks. We use a combination of dry and wet filters. First, the water trickles over the dry filter and is thereby enriched with oxygen. Next, in the wet biofilter, organic matter is degraded and toxic ammonium and nitrite are converted to nitrate. All of the purified water are then exposed to UV light (40 W tubes, changed annually) to eliminate most microorganisms. Finally, the water is further purified by filtration through a mechanical filter that removes particles down to 20 μm diameter. The water is then pumped back into the storage reservoirs at the top of the tank system.

Fresh fish water is added to the system automatically thrice a day, which represents about 5%–10% of the total water volume. To produce fresh fish water, we mix approximately two volumes of demineralized water generated by a centralized demineralization system with one volume of tap water. EZRC is in a privileged situation, since the tap water produced on campus at KIT does not contain chlorine, but is only purified through a sand/gravel filter. Otherwise, chlorine would have to be removed by a carbon filter. Conductivity and pH of the mixed water are continuously measured by probes in the storage tanks. In case of significant deviations, for example, due to temperature and weather conditions (conductivity <200 or >250 μS, pH <6.8 or >7.6), the mix ratio is manually adjusted. Furthermore, pH and temperature in the fish facility are recorded daily. Additional parameters relevant for the water chemistry (NH_4_^+^, NO_2_^−^, NO_3_^−^, PO_4_^3−^, carbonate hardness, and total hardness) are measured and recorded weekly.

Fish are kept at a density of 5 fish/L in the 10 L glass tanks. The water inflow is adjusted to 10 L/h. Three to five snails (*Planorbarius corneus*) are added to each baby tank at 3 weeks postfertilization. Two to eight snails are later transferred to the adult tanks along with the fish. While the use of snails introduces an additional element of complexity in the fish husbandry, it has several advantages as follows: snails improve water quality by taking up leftover food, in particular, in the baby tanks; they remove algae from the tank walls, reducing the need to clean them with the concomitant danger of fish jumping out or being injured; their reproduction in the baby tanks serves as an indicator of adequate feeding; and they are a good indicator for water quality since they are less tolerant than fish to adverse conditions such as high temperatures. The snails that we use are bred by us in designated tanks (mouse cages) in the same fish room, reducing the risk of spreading disease.

In general, tanks are completely cleaned after fish and snails have been removed. Tank walls are scraped and algae and other debris are siphoned out, lids are also cleaned. When maintaining fish in the tanks, every 2 weeks, lids are replaced by clean ones to remove mold growing on food remnants littered by accident when feeding. Every 2 months, tank bottoms are siphoned to remove accumulated debris after walls and bottoms are scraped (if necessary). Water inlet pipes are replaced when algae are growing in them.

We feed our larvae with very fine powder food (such as Caviar 5–50 μm, SAFE) as soon as 70% of the larvae are freely swimming. In our hands, this gives results similar to paramecia. Larvae at 7 days to 1 month of age are fed thrice a day with a 1:1 mix of 5–50 and 50–100 μm powder food, at 1–2 months of age with a 1:2 mix of 100–200 and 200–300 μm powder food and once a day with freshly hatched artemia, and at 2–4 months with a 1:2:1 mix of 200–300, 300–500, and 500–800 μm powder food and once a day with artemia. Adults are fed once a day with a mix of 1 kg granulate feed (such as ST5, Aqua Schwarz): 10 L TetraMin flakes (Tetra; passed through a sieve to reduce flake size) and once with artemia.

## Hygiene Measures

To assure a healthy state of the fish, the EZRC uses a number of handling rules that reduce disease risk and improve the general well-being of the stocks:

• Keep plastic and glassware used in the facility separate from that of the laboratory to avoid contamination of vessels with harmful substances such as detergents or other toxic chemicals.
• Treat fish rooms as isolated units. Do not mix breeding cages, nets, etc. Try to maintain all items within one fish room as much as possible or reintroduce nets, cages, etc., only after appropriate cleaning.
• Minimize reuse of nets etc. that has been in contact with one tank for another tank. Of course, when crossing different strains, direct contact of fish from different tanks cannot be avoided. However, never cross fish from different fish rooms.
• Only introduce eggs into the facility that have been properly bleached. For bleaching, we add 380 μL 12% sodium hypochlorite solution (stored for up to 6 months in a refrigerator) to 1 L of facility water. Six hpf to thirty-six hpf embryos are transferred to a tea strainer and placed for 5 min in bleach, for 5 min in water, again for 5 min in bleach, and for 5 min in water, then transferred to Petri dishes with the E3 medium.
• Never introduce adult fish into the facility or move them between different fish rooms. Always use bleached eggs to move stocks from the quarantine into the facility or between fish rooms.
• Carefully examine the health state of the fish in each tank every day. Remove dead or sick fish immediately. If fish are beyond the reproductive stage (in general, this means after 18 months), terminate them rapidly as they can be a source of diseases.
• The Quarantine room of the EZRC is a totally separate unit with a separate infrastructure for cleaning and food preparation, and in a different building from the Core fish room. Animal care takers have to change shoes and clothing when leaving the quarantine room. They also have to take a shower before entering another fish room.

The following cleaning procedures are used for equipment and rooms:

• Mouse cages and laying cages are cleaned in a commercial dishwasher (e.g., Hobart), using NeoFT (Dr. Weigert) as cleaner. The dishwasher is set to extra rinses with demineralized water. We do not use rinse aid to avoid carrying it over into the fish facility.
• Avoid detergents for cleaning as much as possible. Ensure that there are no remnants left by thoroughly rinsing with demineralized water.
• Nets are rinsed after use and are either autoclaved (in the Core and Quarantine fish rooms) or placed in a water boiler (Rommelsbacher, Dinkelsbühl, with water outlet valve, timer, and temperature controller) at 90°C for a minimum of 20 min.
• We use an autoclave to sterilize cages and nets of the quarantine.
• Avoid hand creams as the oils will wash off and form a film on the water surface.
• Use cleaning solutions for floors etc. that are not harmful for the fish. Our floors are cleaned with freshly diluted 1% Virkon S (DuPont). Bench surfaces and microscopes are cleaned with 80% ethanol.

## Health Monitoring and Genetic Management

All incoming larvae are immediately rinsed, placed in fresh system water, and inspected under a stereomicroscope by a trained animal caretaker for signs of parasites, fungi, and bacteria, and pH and ammonia content of the transport media are recorded. The health of our fish is monitored by an external veterinarian (Fishcare, Abstatt) as follows:

• The Core fish room is fitted with 12 sentinel tanks with AB fish, six tanks each pre- and post filter; three fish per tank are examined every 4 months (starting at the age of 4 months).
• From each of the other fish rooms, six to seven random live fish are examined every 12 months.

Parasitological examination of squash preparations is performed for worms (nematodes, cestodes, trematodes), protozoa (*Ichthyophthirius multifiliis*, Piscinoodinium, Tetrahymena), fungi, and bacteria (such as Mycobacterium, Flavobacterium, Aeromonas). On each fish, an examination of skin, gills, liver, kidney, spleen, gut, and brain is carried out by native preparation/microscopy. If required, H&E staining, PAS, and Ziehl–Neelsen staining are performed on histological sections. In addition, we have begun to use polymerase chain reaction (PCR) and histological analyses carried out by a service company for *Pseudoloma neurophilia* and Mycobacterium, using proprietary protocols, which allow to distinguish several mycobacterial strains (IDEXX Deutschland). We have obtained positive results in some fish for *Pseudoloma neurophilia*, for Flavobacterium sp., for *Mycobacterium chelonae* (considered to be an opportunistic strain), and for an unidentified mycobacterial strain, but not for other pathogens. We follow recommended measures^6^ to mitigate the spread of Pseudoloma infection and where possible, avoid use of the same wild-type fish for outcrossing more than one mutant or transgenic line.

Shipments of embryos are accompanied by a health report from the veterinarian. This report summarizes the results of the tests performed on sentinel fish from the Core fish room in the past years.

Carriers of mutations and transgenes are identified in each generation by allele-specific PCR (KASPar assay; LGC), by morphological phenotyping or by observation of transgene expression, as appropriate for the specific line. Most of the living lines will be terminated eventually once we have preserved them as frozen sperm.

To maintain the complex phenotype of the Sanger lines, the F3 generation used for the preparation of sperm samples is generated from at least four F2 outcrosses, aiming for a total number of 20 adult F3 males to represent the line. For transgenic lines, sperm from as many as 10 identified carriers is pooled and divided into five sperm samples. After successfully testing the obtained samples, the fish are discarded. On average, we grow ∼120 fish to preserve a Sanger line and 60 fish to preserve a transgenic or outcrossed mutant line.

Outcrosses of mutant and transgenic lines are performed against the AB wild-type line, which we originally obtained from the ZIRC. For each generation of AB wild-type fish, 30–60 fish from 10 tanks are set up for single pair mating. To counter a slight reduction in viability that we recently observed, new AB fish were introduced from ZIRC and are being crossed with the AB fish already at the EZRC. We will continue this procedure in the future as necessary.

## Conclusions

Providing a reliable source of zebrafish requires measures on several levels. At the EZRC, we have implemented SOPs for fish handling and laboratory operations, a database that helps to keep track of fish pedigrees and to highlight difficult lines, comprehensive hygiene rules, and cleaning procedures, in particular, strict quarantine rules, genetic management of fish lines, and a program for health assurance that includes regular examinations of sentinel fish by an external veterinarian. Many of these procedures have become part of the curriculum of the International Zebrafish and Medaka Courses (IZMC), which are held several times per year at the KIT (http://izmc.ezrc.kit.edu).

In the near future, targeted genome engineering technologies such as CRISPR-Cas9 are likely to replace many chemical mutagenesis experiments.^7^ This will reduce the requirements for traditional crossing and identification of carriers, since we expect to store reagent libraries and inject eggs on demand. However, the need to provide healthy fish will remain as urgent as ever and we will continue to focus on improving fish care and responding to the needs and concerns of the zebrafish community.

## Notes

^*^ www.eufishbiomed.kit.edu
